# Investigating the Inhibitory Potential of Flavonoids against Aldose Reductase: Insights from Molecular Docking, Dynamics Simulations, and gmx_MMPBSA Analysis

**DOI:** 10.3390/cimb46100683

**Published:** 2024-10-16

**Authors:** Muhammad Yasir, Jinyoung Park, Eun-Taek Han, Jin-Hee Han, Won Sun Park, Wanjoo Chun

**Affiliations:** 1 Department of Pharmacology, Kangwon National University School of Medicine, Chuncheon 24341, Republic of Korea; yasir.khokhar1999@gmail.com (M.Y.); jinyoung0326@kangwon.ac.kr (J.P.); 2 Department of Medical Environmental Biology and Tropical Medicine, Kangwon National University School of Medicine, Chuncheon 24341, Republic of Korea; ethan@kangwon.ac.kr (E.-T.H.); han.han@kangwon.ac.kr (J.-H.H.); 3 Department of Physiology, Kangwon National University School of Medicine, Chuncheon 24341, Republic of Korea; parkws@kangwon.ac.kr

**Keywords:** aldose reductase, molecular docking, molecular dynamic simulation, flavonoids, diabetes mellitus

## Abstract

Diabetes mellitus (DM) is a complex metabolic disorder characterized by chronic hyperglycemia, with aldose reductase playing a critical role in the pathophysiology of diabetic complications. This study aimed to investigate the efficacy of flavonoid compounds as potential aldose reductase inhibitors using a combination of molecular docking and molecular dynamics (MD) simulations. The three-dimensional structures of representative flavonoid compounds were obtained from PubChem, minimized, and docked against aldose reductase using Discovery Studio’s CDocker module. The top 10 compounds Daidzein, Quercetin, Kaempferol, Butin, Genistein, Sterubin, Baicalein, Pulchellidin, Wogonin, and Biochanin_A were selected based on their lowest docking energy values for further analysis. Subsequent MD simulations over 100 ns revealed that Daidzein and Quercetin maintained the highest stability, forming multiple conventional hydrogen bonds and strong hydrophobic interactions, consistent with their favorable interaction energies and stable RMSD values. Comparative analysis of hydrogen bond interactions and RMSD profiles underscored the ligand stability. MMPBSA analysis further confirmed the significant binding affinities of Daidzein and Quercetin, highlighting their potential as aldose reductase inhibitors. This study highlights the potential of flavonoids as aldose reductase inhibitors, offering insights into their binding interactions and stability, which could contribute to developing novel therapeutics for DM complications.

## 1. Introduction

Diabetes mellitus (DM) is a complex and chronic metabolic disorder characterized by persistent hyperglycemia or high blood sugar levels. This condition is associated with significant complications that affect various organs and systems. One key enzyme implicated in the development of these complications is aldose reductase, a member of the aldo-keto reductase superfamily, which plays a pivotal role in the polyol pathway [1,2]. Under normal physiological conditions, aldose reductase has a minimal role in glucose metabolism. However, in the context of DM, elevated blood glucose levels significantly increase its activity, leading to adverse biochemical changes within cells.

Aldose reductase catalyzes the reduction of excess glucose to sorbitol, utilizing nicotinamide adenine dinucleotide phosphate (NADPH) as a cofactor [3,4,5], marking the initial and critical step in the polyol pathway. The accumulation of sorbitol, which cannot easily cross cell membranes, leads to osmotic stress and cellular swelling [6,7]. Furthermore, this conversion process depletes NADPH, a crucial molecule needed for the regeneration of reduced glutathione [8,9,10], one of the cell’s primary antioxidants. The reduction in NADPH availability impairs the cell’s ability to neutralize reactive oxygen species (ROS), contributing to oxidative stress and cellular damage [11,12].

The implications of aldose reductase activity are widespread across various tissues. For instance, in the lens of the eye, sorbitol accumulation is linked to osmotic stress, a key factor in the development of diabetic cataracts [13,14,15]. In peripheral nerves, the combination of oxidative stress and osmotic imbalance leads to a decline in nerve conduction velocity and nerve blood flow, contributing to diabetic neuropathy [16,17,18]. Similar pathogenic mechanisms underlie other severe complications of diabetes, such as diabetic cardiomyopathy, nephropathy, and retinopathy.

Given its central role in these harmful processes, aldose reductase is a critical target for therapeutic intervention. Inhibitors of this enzyme have the potential to alleviate the adverse effects of hyperglycemia on tissues, thereby reducing the risk and severity of diabetic complications [3,19]. This emphasizes the significance of research efforts aimed at identifying and developing effective aldose reductase inhibitors, which offer hope for improving the quality of life for individuals affected by DM and its associated complications.

Moreover, herbs have a longstanding history of use in traditional medicine and encompass a diverse array of phytochemical components, such as terpenoids, phenols, lignins, stilbenes, tannins, flavonoids, quinones, coumarins, alkaloids, amines, betalains, and various other metabolites [20]. Among these, flavonoids stand out as low molecular weight phenolic compounds found abundantly in numerous plant species [21]. Flavonoids are renowned for their wide-ranging biological properties, including anti-cancer, anti-inflammatory, antioxidant, anti-mutagenic, anti-allergic, and anti-viral activities [22,23]. Previously, a study explored Acumitin and Agathisflavone as potent flavonoid compounds against aldose reductase, primarily focusing on molecular docking studies [24].

**Figure 1:**
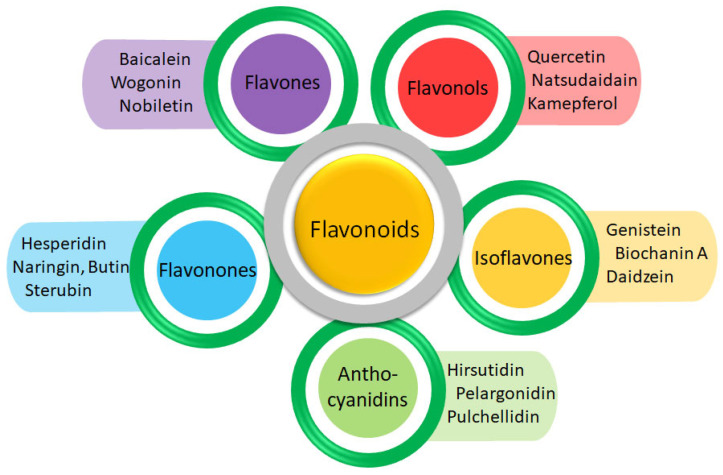
The representative compounds from the respective flavonoid subfamily.

This study aims to explore the potential of flavonoids against aldose reductase. By targeting aldose reductase, flavonoids may help mitigate these complications by reducing sorbitol accumulation and oxidative stress, thereby preserving nerve conduction velocity, improving nerve blood flow, and potentially alleviating symptoms associated with diabetic neuropathy [25,26]. This study enhances the current knowledge of flavonoids in diabetes management by offering detailed molecular insights, bridging the gap between existing experimental evidence and potential therapeutic applications. The unique group of structurally diverse flavonoid compounds across different subclasses (Figure 1) emphasizes the potential of flavonoid compounds as promising aldose reductase inhibitors. Identifying flavonoids that exhibit strong binding affinity and favorable interactions with aldose reductase could pave the way for developing novel treatments or supplements to complement existing diabetic management strategies.

## 2. Materials and Methods

### 2.1. Aldose Reductase Structure Retrieval

The three-dimensional structure of human aldose reductase (PDB ID: 1 PWM, 0.92 Å resolution bound to NADP and Fidarestat) was obtained from the Protein Data Bank (https://www.rcsb.org/ (accessed on 10 October 2024)). Energy minimization receptor preparations were performed using UCSF Chimera v1.16 and Discovery Studio Client v22 [27,28]. (See Supplementary Data Section S1).

### 2.2. Prediction of Active Binding Site

The bound ligand (Fidarestat) was selected for the binding pocket generation, and the binding sphere was created using the Define Binding Site window in Discovery Studio. To enhance docking accuracy, the binding sphere was refined with constraints specific to the selected amino acids (See Supplementary Data Section S2).

### 2.3. Molecular Docking

Molecular docking is a widely used method for evaluating ligand–receptor interactions. It predicts the binding strength or binding energy of protein–ligand complexes by analyzing their preferred orientations using scoring algorithms [29,30]. The protein was prepared by removing the already bound ligand and water molecules, followed by adding hydrogen atoms using Discovery Studio’s receptor preparation module. Ligand preparations for candidate compounds involved generating tautomers, adjusting ionization states, and correcting any valence issues, utilizing the Ligands Preparation module in Discovery Studio Client v22. Molecular docking of the ligands against the target protein, aldose reductase, was performed using the CDocker module in Discovery Studio with default orientation and conformation settings. The best-docked complexes were evaluated based on the lowest docking energy values, measured in kcal/mol.

### 2.4. MD Simulations

The top compounds with the lowest docking energy were selected for a 100 ns MD simulation. The protocols for the MD simulation experiment were adapted from our previously published papers [31,32]. The CHARMM36 force field was set up using the solution builder protocol on the CHARMM-GUI server (https://www.charmm-gui.org/?doc=input/solution (accessed on 10 October 2024)). This interface was also used to generate input files for MD simulations with GROMACS 2019.3 [33].

The system was solvated using the TIP3P-3 point water model in a cubic box with periodic boundary conditions. Neutralization was achieved by adding counter ions. Electrostatic and van der Waals interactions were calculated using the Verlet method with a 10 Å cut-off radius, and the LINCS algorithm was employed to constrain the bond lengths during simulations. Additionally, accurate electrostatic interactions were computed using the Particle Mesh Ewald (PME) approach. The solvated systems were prepared using the steepest descent energy minimization method. Two equilibration phases were then conducted: first under constant temperature, constant volume (NVT) conditions, followed by constant temperature, constant pressure (NPT) conditions. A built-in Python script from CHARMM-GUI was used to convert the GROMACS topology (top) and parameter (itp) files for the MD simulations. Structural analysis of the protein–ligand complexes was performed using GROMACS v2019.3 on a Linux platform [34]. Therefore, a 2 fs time step was employed to run the MD simulations in GROMACS.

### 2.5. gmxMMPBSA Binding Free Energy Calculation

A program gmx_MMPBSA v1.6.3 was developed to compute the end state-free energies of protein–ligand complexes from GROMACS MD trajectory data [35]. Binding free energy predictions were made using an MM/PBSA approach from the MD simulation trajectories in explicit solvent, analyzing the three components, such as the complex, receptor, and ligand, separately [36]. The binding free energy (ΔG_binding_) of the lead compounds in complex with the protein was determined using the following equation:

ΔG_binding_ = G_complex_ − (G_protein_ + G_ligand_) (1)

In this equation, G_complex_ represents the energy of the lead compound–protein complexes, and G_protein_ and G_ligand_ demonstrate the proteins’ and ligands’ energy in an aqueous environment, respectively.

## 3. Results and Discussion

### 3.1. Structural Analysis of the Aldose Reductase Protein

The aldose reductase enzyme consists of a single chain composed of 316 amino acids. The protein’s architecture includes α-helices, β-sheets, and coils. VADAR 1.8 statistical analysis indicates that the protein comprises approximately 35% α-helices, 24% β-sheets, 40% coils, and 27% turns. Ramachandran plots show that 98% of the residues are located in favored regions, 99.8% are in allowed regions, with a single outlier (Glu84) for the dihedral angles phi (φ) and psi (ψ) (Supplementary Data Section S1).

### 3.2. The Binding Pocket Analysis

Using Discovery Studio’s ligand interaction method, the binding pocket residues of aldose reductase were identified as Val47, Trp111, Trp79, His110, Tyr48, Trp20, Trp219, Cys298, Ala299, Leu300, and Phe122. These residues were further validated against the existing published data [37]. To investigate the accurate interaction of flavonoid compounds within the active site of aldose reductase, the binding sphere coordinates were set to X = 22.9571, Y = 1.0560, and Z = 34.0032, with a radius of 8.2904, based on the binding pocket residues (Supplementary Data Section S2).

### 3.3. Ligands Preparation

Aldose reductase has a smaller and more hydrophobic active site, which requires inhibitors to be designed with higher specificity to effectively block its activity without affecting the related enzymes. Unlike traditional ANSAID, which often targets complex active sites of enzymes like COX-1 and COX-2, flavonoids are particularly well-suited to interact with the smaller and more hydrophobic active site of aldose reductase. Moreover, the specificity of flavonoids in targeting aldose reductase without affecting the related enzymes minimizes the risk of off-target effects, which is a common concern with synthetic inhibitors.

**Figure 2:**
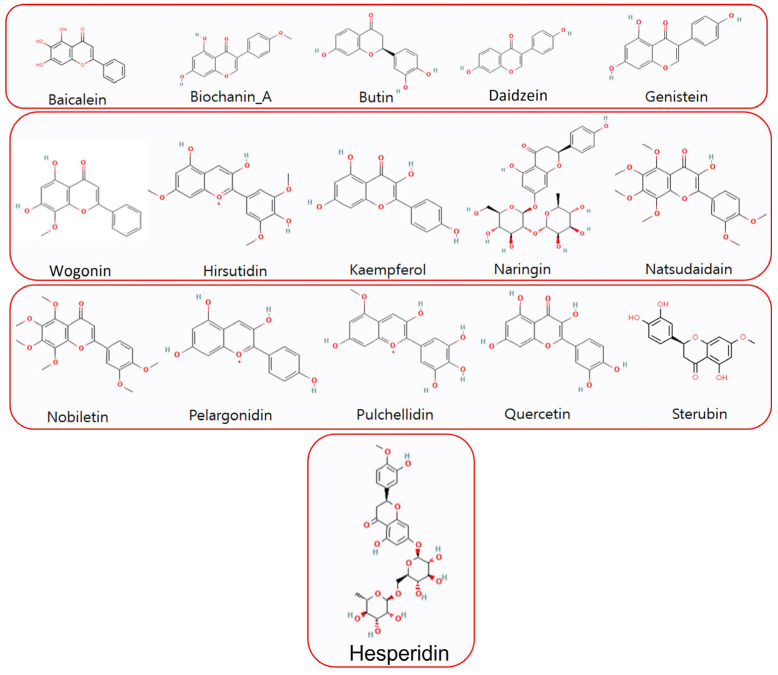
The structural assessment of 2D structures of screened flavonoids for molecular docking.

Flavonoids possess various beneficial properties, including anti-cancer, antioxidant, anti-allergic, and anti-inflammatory effects, making them effective against a range of diseases [22,38]. The 3D structures of representative flavonoid compounds from different subclasses of flavonoids were acquired from PubChem based on their recent anti-diabetic biological activities (Table 1). These structures were further optimized using Discovery Studio and UCSF Chimera v1.16. Following the structural analysis (both 2D and 3D), the most promising ligands were selected for subsequent molecular docking studies (Figure 2).

### 3.4. Molecular Docking Analysis

The CDocker module in Discovery Studio was used to predict two types of energy values: CDocker energy and CDocker interaction energy. CDocker energy reflects the overall docking energy considering the 3D structural and physicochemical properties of both the ligand and protein. On the other hand, CDocker interaction energy specifically measures the energy associated with interactions between the ligand and the receptor. This includes contributions from various intermolecular forces such as van der Waals forces, electrostatic interactions, and hydrogen bonding, collectively influencing the binding affinity. CDocker interaction energy provides detailed insights into the strength and nature of specific interactions between the ligand and the receptor [75].

Daidzein exhibited a CDocker energy of −41.0403 kcal/mol and a CDocker interaction energy of −42.9466, indicating strong overall docking energy and favorable interaction with the enzyme. Quercetin showed a CDocker energy of −37.6379 and a CDocker interaction energy of −47.167, demonstrating substantial binding affinity and interaction strength. Kaempferol recorded a CDocker energy of −34.4646 and a CDocker interaction energy of −44.7002, suggesting good binding properties and interaction potential. Butin, with a CDocker energy of −31.0165 and a CDocker interaction energy of −36.0224, also showed notable docking and interaction characteristics (Table 2). Naringin and hesperidin exhibited positive docking energy values. While their interactions are comparable, the overall CDocker energy is high, indicating the lowest compatibility with aldose reductase.

The results indicate that the top flavonoids, particularly Daidzein, Quercetin, and Kaempferol, possess strong binding and interaction energies with aldose reductase. This suggests that these compounds might be effective in inhibiting the enzyme’s activity, potentially mitigating the harmful effects of hyperglycemia in diabetic complications. The low CDocker interaction energy values of these compounds reflect their strong and specific interactions with the active site of aldose reductase, which could translate to effective inhibition. Previously, a study explored Acumitin and Agathisflavone as promising aldose reductase inhibitors, primarily focusing on molecular docking studies [24]. They found that these compounds formed more interactions against the key residues of aldose reductase. Therefore, to analyze the stability of the docked compounds over time, it is important to carry out MD simulations. Therefore, we choose the top 10 docked flavonoid compounds for MD simulation.

### 3.5. Molecular Dynamics (MD) Simulations

To assess the stability of the screened compounds against aldose reductase, the docked complexes were subjected to MD simulations using GROMACS. These simulations were carried out for a duration of 100 ns to investigate the behavior and stability of the complexes over time.

#### 3.5.1. Root Mean Square Deviation

The root mean square deviation (RMSD) analysis of the flavonoid compounds provides key insights into their stability when bound to aldose reductase. RMSD is a crucial parameter in assessing the stability of the ligand–receptor complex during MD simulations, with lower RMSD values generally indicating greater stability and more reliable binding interactions (Supplementary Data Figure S4).

**Figure 3:**
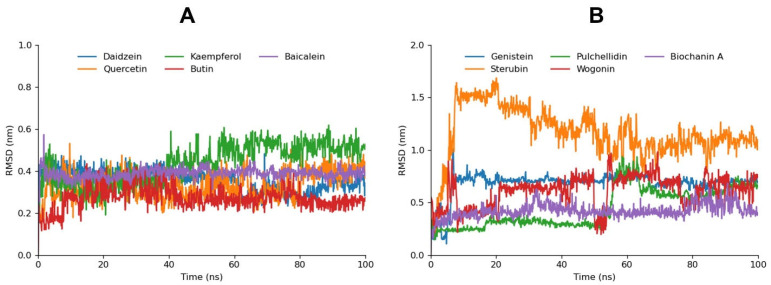
Graph (**A**) depicts the RMSD bar graphs for Daidzein, Kaempferol, Baicalein, Quercetin, and Butin. Graph (**B**) displays the RMSD bar graphs for Genistein, Pulchellidin, Biochanin A, Sterubin, and Wogonin.

Daidzein, Quercetin, and Baicalein displayed notably stable RMSD values, which remained consistent throughout the simulation. This stability suggests that these compounds maintain a strong and stable binding conformation within the aldose reductase active site, reinforcing the results from the molecular docking analysis, where these compounds also showed strong binding affinities. Butin exhibited relatively low RMSD values as well, indicating good stability, while Kaempferol, despite a slight increase in the RMSD after 40 ns, maintained overall stability, suggesting that its binding conformation remains largely intact with minimal fluctuations. This slight increase in RMSD may indicate minor adjustments in the ligand’s position within the binding pocket but does not significantly detract from its overall binding stability (Figure 3). Genistein, which ranked fifth in the molecular docking analysis, showed stable but comparatively higher RMSD values. The higher RMSD indicates more pronounced conformational adjustments or flexibility within the binding pocket. However, this does not necessarily undermine its binding affinity but rather suggests a dynamic interaction, which can still be favorable after the ligand’s structural optimization.

In contrast, compounds like Sterubin, Pulchellidin, Biochanin A, and Wogonin exhibited relatively higher RMSD fluctuations. These fluctuations suggest that these ligands may not maintain a stable binding conformation, possibly due to weaker interactions with the active site or a lack of key stabilizing contacts. The higher RMSD values for these compounds correlate with their lower docking scores, indicating that both their binding affinity and stability are less favorable.

The correlation between RMSD fluctuations and ligand stability is evident from this analysis. Compounds with strong docking affinities that also maintain low and stable RMSD values are more likely to form robust, reliable interactions with aldose reductase, making them better candidates as potential inhibitors. On the other hand, higher RMSD fluctuations, even in the presence of moderate docking scores, could indicate less favorable binding dynamics.

#### 3.5.2. Hydrogen Bond Plot

**Figure 4:**
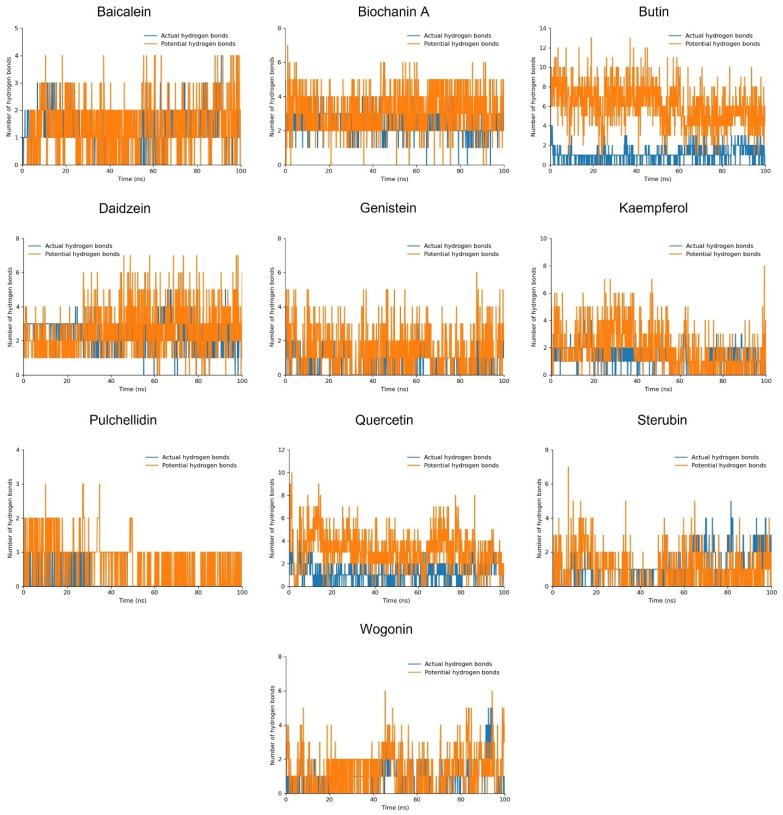
The hydrogen bond plot of the simulated flavonoid compounds.

Daidzein and Quercetin formed two actual hydrogen bonds and consistently maintained two potential hydrogen bonds, with the peaks indicating the possibility of forming additional hydrogen bonds. Butin, Kaempferol, and Baicalein also established one consistent actual hydrogen bond along with two or three potential hydrogen bonds, with the peaks suggesting up to ten potential hydrogen bonds. Biochanin A, Genistein, Sterubin, and Wogonin maintained one actual hydrogen bond and several potential hydrogen bonds. Despite the relatively lower overall stability of these compounds, as indicated by the RMSD analysis, their interaction profiles were sustained throughout the 100 ns MD simulation (Figure 4).

Comparing these hydrogen bond interactions with the RMSD results, we observe that compounds like Daidzein and Quercetin, which have strong docking scores and stable RMSD values, also maintain multiple hydrogen bonds consistently. This suggests that their stable binding interactions contribute to their overall structural stability. On the other hand, Biochanin A, Genistein, Sterubin, and Wogonin, while showing consistent hydrogen bond interactions, have higher RMSD fluctuations. This indicates that their overall binding stability is less reliable.

#### 3.5.3. MD Interaction Energy

The interaction energies of various flavonoid compounds with aldose reductase were analyzed, focusing on Coulomb–SR, Lennard–Jones–SR, and the total interaction energy (Table 3). Daidzein exhibited the highest total interaction energy at −201.3170 KJ/mol, with significant contributions from both the Coulomb–SR (−84.11102 KJ/mol) and Lennard–Jones–SR (−117.2060 KJ/mol) interactions, indicating strong and stable binding with the enzyme. Quercetin, with a total interaction energy of −159.8031 KJ/mol, showed a substantial Lennard–Jones–SR interaction (−123.0920 KJ/mol), highlighting its favorable binding characteristics. Kaempferol displayed a total interaction energy of −167.8917 KJ/mol, supported by the robust Lennard–Jones–SR interaction (−128.0307 KJ/mol), suggesting strong binding affinity.

**Figure 5:**
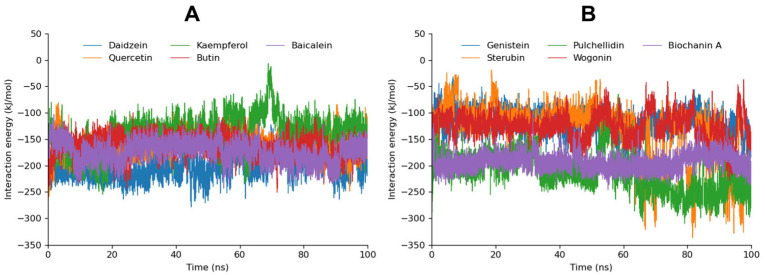
The calculated interaction energy of Daidzein, Quercetin, Kaempferol, Butin, and, Baicalein is depicted in graph (**A**) while the interaction energy of Genistein, Pulchellidin, Sterubin, Wogonin, and Biochanin A is manifested in graph (**B**) during the 100 ns MD trajectory.

Baicalein, with a total interaction energy of −173.6471 KJ/mol, showed strong binding affinity, particularly due to its substantial Lennard–Jones–SR interaction (−130.754 KJ/mol). Pulchellidin displayed a total interaction energy of −152.7948 KJ/mol, with a significant Coulomb–SR interaction (−66.2548 KJ/mol). Wogonin had a total interaction energy of −126.5103 KJ/mol, with moderate interaction contributions. Biochanin A exhibited a total interaction energy of −154.3663 KJ/mol, with a notable Lennard–Jones–SR interaction (−93.4600 KJ/mol). The interaction energy over a 100 ns MD trajectory is graphically represented in Figure 5. The individual graphs for each compound are depicted in Supplementary Data Figure S5.

Comparing these interaction energies with previous RMSDs and hydrogen bond analyses, it is evident that compounds such as Daidzein, Quercetin, and Kaempferol, which demonstrated strong binding affinities and stable interaction profiles, are corroborated by their low total interaction energies. These compounds not only maintain stable RMSD values but also exhibit strong Coulombic and van der Waals interactions, reinforcing their potential effectiveness as aldose reductase inhibitors. On the other hand, compounds like Genistein and Sterubin, despite lower total interaction energies, still showed consistent hydrogen bonding and moderate RMSD stability, indicating their potential but less robust binding stability. The higher fluctuations in RMSD values for compounds like Pulchellidin, Wogonin, and Biochanin A, despite their interaction energies, suggest that while they can form significant interactions, their overall binding stability may be less reliable.

#### 3.5.4. Binding at 100 ns

**Figure 6:**
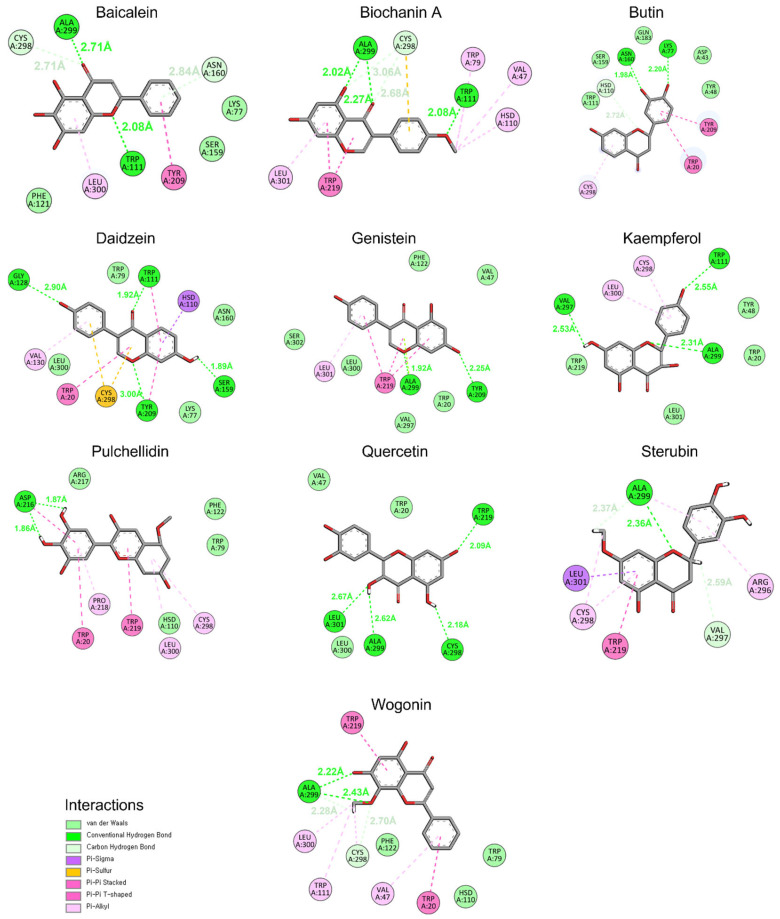
The conformation and interaction analysis of simulated compounds at 100 ns MD simulation.

To confirm the stability of flavonoids against aldose reductase, snapshots at 100 ns of MD simulation were captured and analyzed using Discovery Studio. The interaction profile revealed that the ligands remained docked to the active site amino acids of aldose reductase, maintaining specific interactions throughout the simulation (Figure 6). Daidzein and Quercetin each formed four conventional hydrogen bonds and exhibited additional hydrophobic interactions, indicating strong and stable binding. Conventional hydrogen bonds involve a hydrogen atom bonded to an electronegative atom that interacts with another electronegative atom. These bonds are relatively strong as compared to carbon–hydrogen bonds. Carbon–hydrogen bonds, on the other hand, involve a hydrogen atom bonded to a carbon atom interacting with an electronegative atom. Kaempferol formed three conventional hydrogen bonds along with other hydrophobic interactions, demonstrating substantial binding affinity. Baicalein formed two conventional hydrogen bonds and two carbon–hydrogen bonds, suggesting a balanced interaction profile. Biochanin A displayed three conventional hydrogen bonds and two carbon–hydrogen bonds, showing a mix of strong and moderate interactions. In contrast, Butin, Genistein, Sterubin, Pulchellidin, and Wogonin each formed one or two conventional hydrogen bonds, indicating relatively weaker interactions.

Daidzein and Quercetin, which formed multiple conventional hydrogen bonds, exhibited both high interaction energy and stable RMSD values. Kaempferol, with its three conventional hydrogen bonds, also showed strong binding, aligning with its favorable docking and RMSD results. Baicalein and Biochanin A, with their mix of conventional and carbon–hydrogen bonds, demonstrated moderate stability and binding strength, corroborating their interaction energy profiles. The weaker binding observed for Butin, Genistein, Sterubin, Pulchellidin, and Wogonin, which formed fewer conventional hydrogen bonds, is consistent with their higher RMSD fluctuations and lower overall interaction energies.

### 3.6. Binding Free Energy Calculation

**Figure 7:**
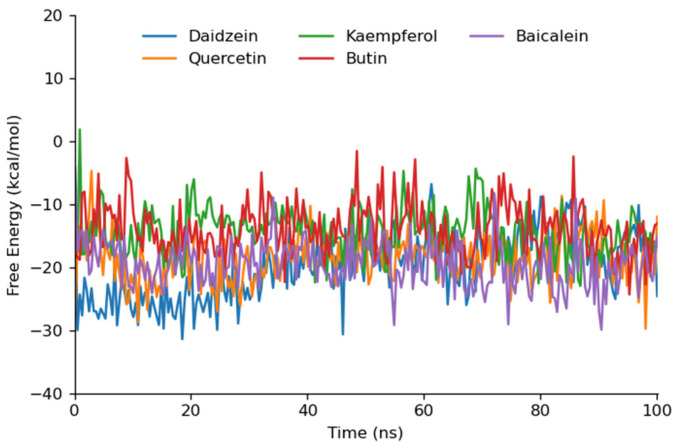
The graphical depiction of gmx_MMPBSA free energy trajectory of top 5 simulation best compounds.

The binding free energy for Daidzein, Quercetin, Kaempferol, Butin, and Baicalein, which exhibited good stability profiles in the 100 ns MD simulations, was calculated using the entire MD trajectory data. The MD trajectory was divided into five segments, and the free energy was calculated for each segment individually to provide detailed MMPBSA insights. The gmx_MMPBSA tool of GROMACS and the MM/PBSA method with default parameters were employed to compute the binding energy (Figure 7).

The free energy calculations for the flavonoid compounds against aldose reductase were determined, with the results summarized in terms of average ΔG and standard deviation. Quercetin exhibited the most favorable free energy of binding with an ΔG of −19.74 kcal/mol, indicating a strong binding affinity. Diadzein followed closely with an ΔG of −19.68, also suggesting a high binding potential. Baicalein demonstrated a comparable binding affinity with an ΔG of −19.22, while Butin and Kaempferol showed slightly lower affinities with ΔG values of −16.28 and −15.65, respectively (Table 4). The lower average ΔG values for Quercetin, Daidzein, and Baicalein correlate with their stable interaction profiles observed during the MD simulations. These compounds formed multiple conventional hydrogen bonds, contributing to their higher binding affinities. Butin and Kaempferol, although showing slightly higher ΔG values, still demonstrated significant binding interactions, supported by their RMSD stability and hydrogen bonding patterns (Supplementary Data Figure S6). Overall, the free energy data supports the molecular docking and MD simulation results, highlighting Daidzein and Quercetin as promising aldose reductase inhibitors in our computational study. The stability and strength of these interactions suggest that these flavonoid compounds could be effective in mitigating the effects of hyperglycemia and preventing diabetic complications.

## 4. Conclusions

This study systematically evaluated the inhibitory potential of several flavonoid compounds against aldose reductase, a pivotal enzyme involved in diabetic complications. Through a combination of molecular docking, MD simulations, and MMPBSA free energy calculations, we identified Daidzein and Quercetin as the top candidates, demonstrating strong binding interactions, stable hydrogen bonding profiles, and favorable binding free energies. Kaempferol, Butin, and Baicalein also showed significant binding affinities, though with slightly lower stability in comparison to the leading compounds. Importantly, the detailed analysis of the interaction energies and stability profiles revealed key insights into the compatibility of flavonoids with the smaller, hydrophobic binding pocket of aldose reductase.

This work extends the current understanding of flavonoids in diabetes management by providing in-depth molecular insights that bridge the gap between existing experimental evidence and potential therapeutic applications. The findings not only underscore the relevance of flavonoid compounds as promising aldose reductase inhibitors but also pave the way for their further optimization. While the results present promising computational evidence, future studies will be crucial to confirm their efficacy in clinical contexts. Therefore, this study serves as a critical foundation for guiding experimental validation and the rational design of novel therapeutics to mitigate diabetic complications.

## Figures and Tables

**Table 1:** The molecular weight and the target diseases of the selected flavonoid compounds.

Compounds	Mol Weight g/mol	Target Disease	Reference
Daidzein	254.2	DM	[39,40,41]
Quercetin	302.2	DM	[42,43,44]
Genistein	270.2	DM	[45,46]
Kaempferol	286.2	DM	[47,48,49]
Butin	272.2	Ischemia, DM	[50,51,52]
Sterubin	302.3	Alzheimer’s disease, DM	[53,54]
Baicalein	270.2	DM	[55,56,57]
Pulchellidin	317.3	DM	[58]
Wogonin	284.3	DM	[59,60]
Biochanin A	284.3	DM	[61,62]
Pelargonidin	306.7	DM	[63,64]
Natsudaidain	418.4	Antioxidant, Antihyperglycemic	[65]
Hirsutidin	345.3	Parkinson’s Disease, DM	[66,67]
Nobiletin	402.4	DM	[68,69]
Naringin	580.5	DM	[70,71]
Hesperidin	610.6	DM	[72,73,74]

**Table 2:** The docking energy values (kcal/mol) of flavonoids against aldose reductase were calculated by Discovery Studio.

Compounds	CDocker Energy (kcal/mol)	CDocker Interaction Energy (kcal/mol)
Daidzein	−41.0403	−42.9466
Quercetin	−37.6379	−47.1670
Kaempferol	−34.4646	−44.7002
Butin	−31.0165	−36.0224
Genistein	−30.3677	−35.1992
Sterubin	−29.5728	−37.2468
Baicalein	−29.4959	−31.2928
Pulchellidin	−27.2066	−43.7286
Wogonin	−22.8362	−31.4787
Biochanin A	−22.4452	−34.6934
Pelargonidin	−19.4438	−38.2605
Natsudaidain	−14.8916	−54.1690
Hirsutidin	−13.4281	−44.4850
Nobiletin	−10.6368	−47.4056
Naringin	0.6767	−49.4979
Hesperidin	11.1379	−46.3707

**Table 3:** The calculated interaction energy of simulated compounds both in Coulomb–SR and Lennard–Jones–SR. The calculated sum of both is total energy.

Sr No.	Compound	Interaction Energy (KJ/mol)		
		Coul-SR	LJ-SR	Total Energy
1	Daidzein	−84.1110	−117.2060	−201.3170
2	Quercetin	−36.7111	−123.0920	−159.8031
3	Kaempferol	−39.8610	−128.0307	−167.8917
4	Butin	−44.9313	−116.6480	−161.5793
5	Genistein	−24.2369	−93.9965	−118.2334
6	Sterubin	−52.7002	−85.8427	−138.5429
7	Baicalein	−42.8931	−130.7540	−173.6471
8	Pulchellidin	−66.2548	−86.5400	−152.7948
9	Wogonin	−31.6840	−94.8263	−126.5103
10	Biochanin A	−60.9063	−93.4600	−154.3663

**Table 4:** The MMPBSA binding free energy of the top 5 simulated compounds in every 20 ns simulation.

Sr No.	Compound	1 ns–20 ns		20 ns–40 ns		40 ns–60 ns		60 ns–80 ns		80 ns–100 ns		Average	
		ΔG	SD	ΔG	SD	ΔG	SD	ΔG	SD	ΔG	SD	ΔG	SD
**1**	Daidzein	−24.82	3.32	−22.59	3.88	−19.40	3.55	−15.87	3.72	−15.73	4.24	−19.68	3.74
**2**	Quercetin	−18.26	5.05	−20.94	3.71	−18.45	2.88	−19.86	3.12	−21.18	4.10	−19.74	3.77
**3**	Kaempferol	−14.18	4.18	−18.09	3.06	−16.86	3.04	−13.49	3.74	−15.61	4.05	−15.65	3.61
**4**	Butin	−18.15	3.31	−16.52	3.23	−16.64	3.98	−14.32	4.00	−15.80	4.10	−16.28	3.72
**5**	Baicalein	−18.18	4.50	−18.49	2.72	−19.07	2.52	−20.07	3.57	−20.33	3.57	−19.22	3.37

## Data Availability

The data that support the findings of this study are available from the corresponding author upon reasonable request.

## Supplementary Materials

The following supporting information can be downloaded at: https://www.mdpi.com/article/10.3390/cimb46100683/s1, References [76,77,78,79,80,81,82,83,84,85] are cited in the supplementary materials file.
