# Supplementary material for: Investigating the Inhibitory Potential of Flavonoids against Aldose Reductase: Insights from Molecular Docking, Dynamics Simulations, and gmx_MMPBSA Analysis
Source: Curr Issues Mol Biol. 2024 Oct 16;46(10):11503–18. doi: 10.3390/cimb46100683 (PMC11506312; doi:10.3390/cimb46100683)
Supplement: Supplementary file 1 [file cimb-46-00683-s001.zip › cimb-3243609-supplementary.pdf]

Supplementary data

Investigating the Inhibitory Potential of Flavonoids Against Aldose Reductase: Insights from Molecular Docking, Dynamics Simulations, and gmx_MMPBSA Analysis

Muhammad Yasir [1], Jinyoung Park [1], Eun-Taek Han [2], Jin-Hee Han [2], Won Sun Park [3] and Wanjoo Chun [1,*]

[1]Department of Pharmacology, Kangwon National University School of Medicine, Chuncheon, 24341, Republic of Korea

[2]Department of Medical Environmental Biology and Tropical Medicine, Kangwon National University School of Medicine, Chuncheon, 24341, Republic of Korea

[3]Department of Physiology, Kangwon National University School of Medicine, Chuncheon, 24341, Republic of Korea

*Correspondence: Dr. Wanjoo Chun, Department of Pharmacology Kangwon National University School of Medicine, Kangwon National University, Email: wchun@kangwon.ac.kr, Phone: +82-33-250-8853

1. Structural of Aldose reductase

Aldose reductase is an enzyme belonging to the aldo-keto reductase (AKR) superfamily, which also includes members like prostaglandin G/H synthase 1 (PGHS-1) and prostaglandin G/H synthase 2 (PGHS-2), commonly known as COX-1 and COX-2, respectively [1, 2]. Unlike COX-1 and COX-2, which are involved in prostaglandin synthesis, aldose reductase primarily catalyzes the reduction of glucose to sorbitol, playing a key role in the polyol pathway and being implicated in diabetic complications [3].

This protein includes alpha-helices, beta-sheets, coils, and turns, was subjected to detailed quantitative structural analysis using the VADAR internet server (http://vadar.wishartlab.com/) (Figure S2) [4]. The graphical depiction of Ramachandran's Plot was performed using Discovery Studio (Figure S1).

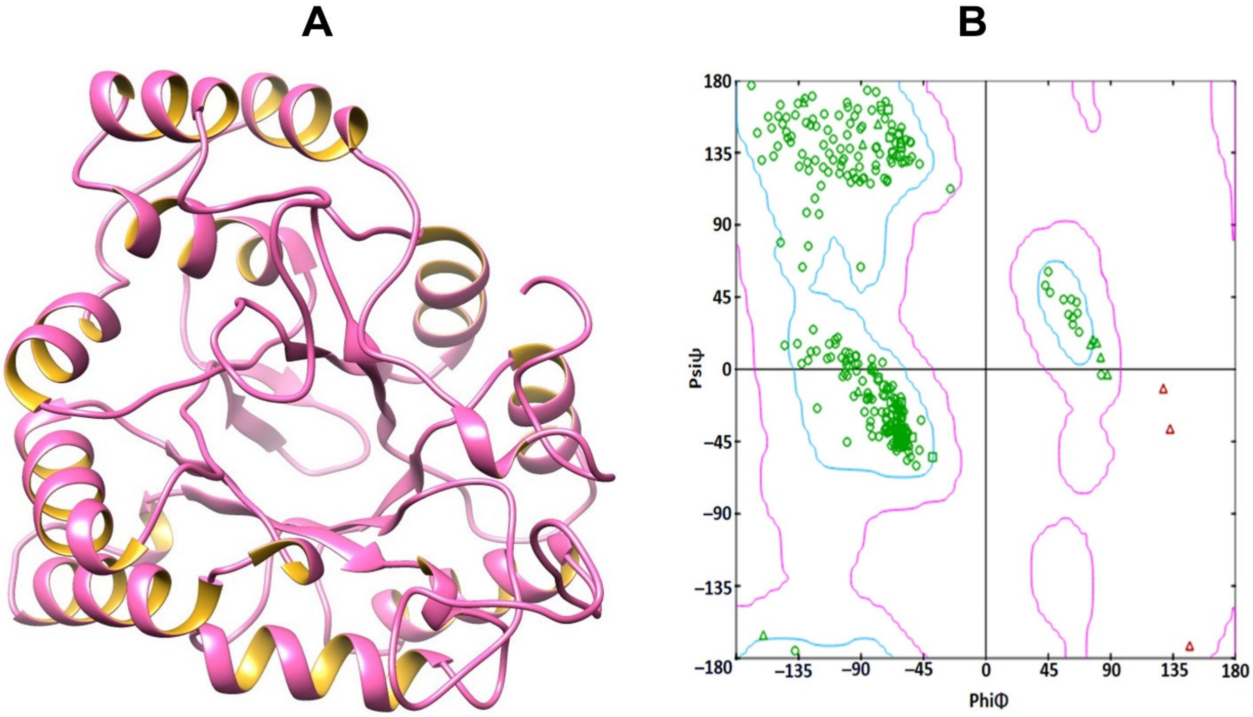

Figure S1. (A, B). The three-dimensional structure of the aldose reductase and the Discovery Studio computed Ramachandran plot (B).

```
**********************************
*            VADAR STATS         *
**********************************
** Using atomic radii from Shrake **
```

(The expected values represent those numbers which would be expected
for highly refined Xray and NMR protein structures.
See the help pages for more information on expected values.)

Statistic	Observed	Expected
# Helix	112 (35%)	-
# Beta	76 (24%)	-
# Coil	128 (40%)	-
# Turn	88 (27%)	-

HYDROGEN BONDS (hbonds)

Statistic	Observed	Expected
Mean hbond distance	2.2 sd=0.4	2.2 sd=0.4
Mean hbond energy	-1.8 sd=1.1	-2.0 sd=0.8
# res with hbonds	247 (78%)	237 (75%)

Expected values obtained from Morris AL, MacArthur MW, Hutchinson EG and Thornton JM. Proteins. 1992 Apr;12(4):345-364.

Figure S2. The statical values of VADAR.

2. Binding Pocket identification

The functionality of a binding pocket is determined by its shape, position within the protein, and the arrangement of surrounding amino acid residues which play an important part in the interaction [5, 6]. When comparing aldose reductase to other members of the prostaglandin G/H synthase family, it is important to note the structural and functional differences that can impact the binding of ligands. COX enzymes, for instance, have larger and more complex active sites, which are often targeted by nonsteroidal anti-inflammatory drugs (NSAIDs). These drugs interact with the cyclooxygenase active site, inhibiting the conversion of arachidonic acid to prostaglandins [7-9]. In contrast, aldose reductase has a smaller and more hydrophobic active site, which requires inhibitors to be designed with higher specificity to effectively block its activity without affecting related enzymesThe binding pocket of a protein is most likely determined by the position of a ligand within the protein's holo-structure [10].

The aldose reductase and inhibitor (NADP and fidarestat) complex (PDB ID: 1PWM) was obtained from the Protein Data Bank (PDB). Interacting amino acids were identified using Discovery Studio's ligand interaction method for the insurance of precise binding site generation. The bound ligand was then selected, and the binding sphere was created using the Define Binding Site window in Discovery Studio. To enhance docking accuracy, the binding sphere was refined with constraints specific to the selected amino acids. Using Discovery Studio's ligand interaction method, the binding pocket residues of aldose reductase were

identified as Val47, Trp111, Trp79, His110, Tyr48, Trp20, Trp219, Cys298, Ala299, Leu300, and Phe122. These residues were further validated against existing published data [11]. To investigate the accurate interaction of flavonoid compounds within the active site of aldose reductase, the binding sphere coordinates were set to X=22.9571, Y=1.0560, and Z=34.0032, with a radius of 8.2904, based on the binding pocket residues (Figure S3).

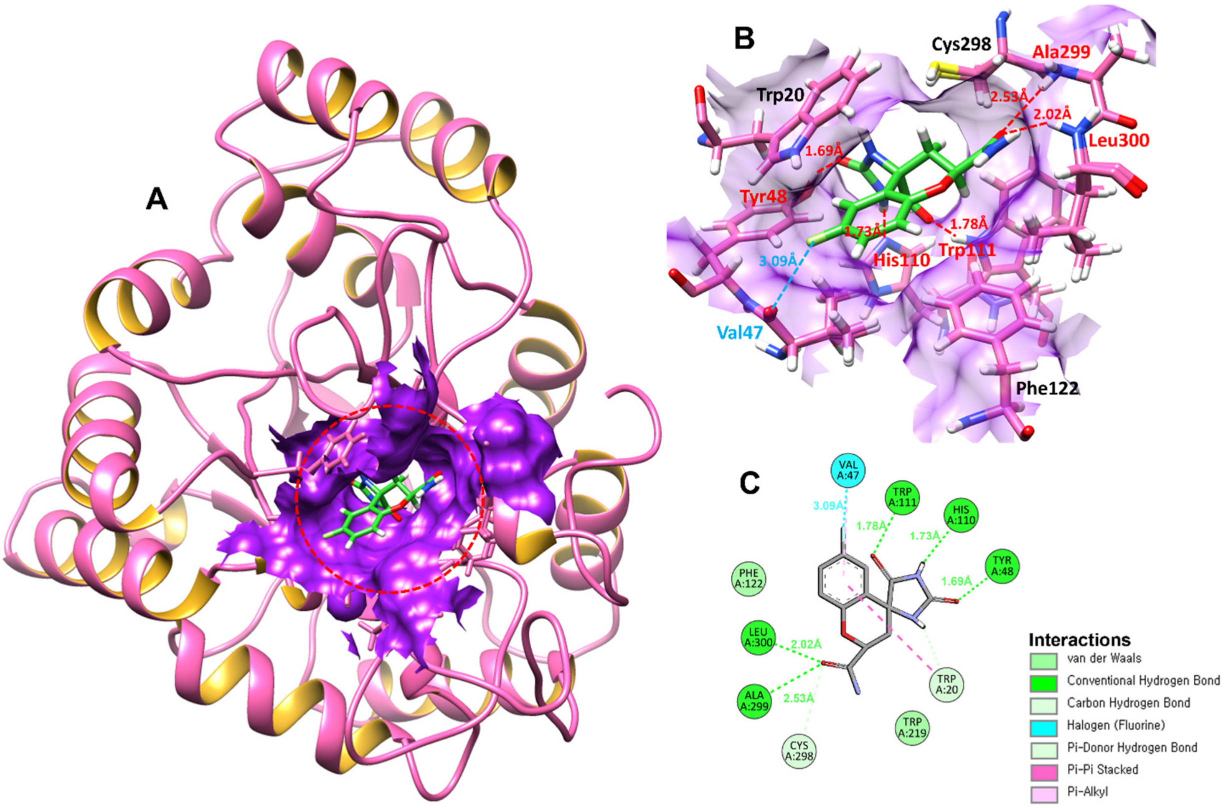

Figure S3. (A, B, C). The figure illustrates the binding pocket of aldose reductase. The ribbon structure is shown in hot pink, with golden rods representing the interior regions, and the binding surface area highlighted in purple (A). The active site residues are marked in the active region of the target protein, where the bound ligand (green) interacts with the receptor (B). Additionally, the interactions between the ligand (Fidarestat) and aldose reductase are depicted using various colors to indicate different interaction types (C).

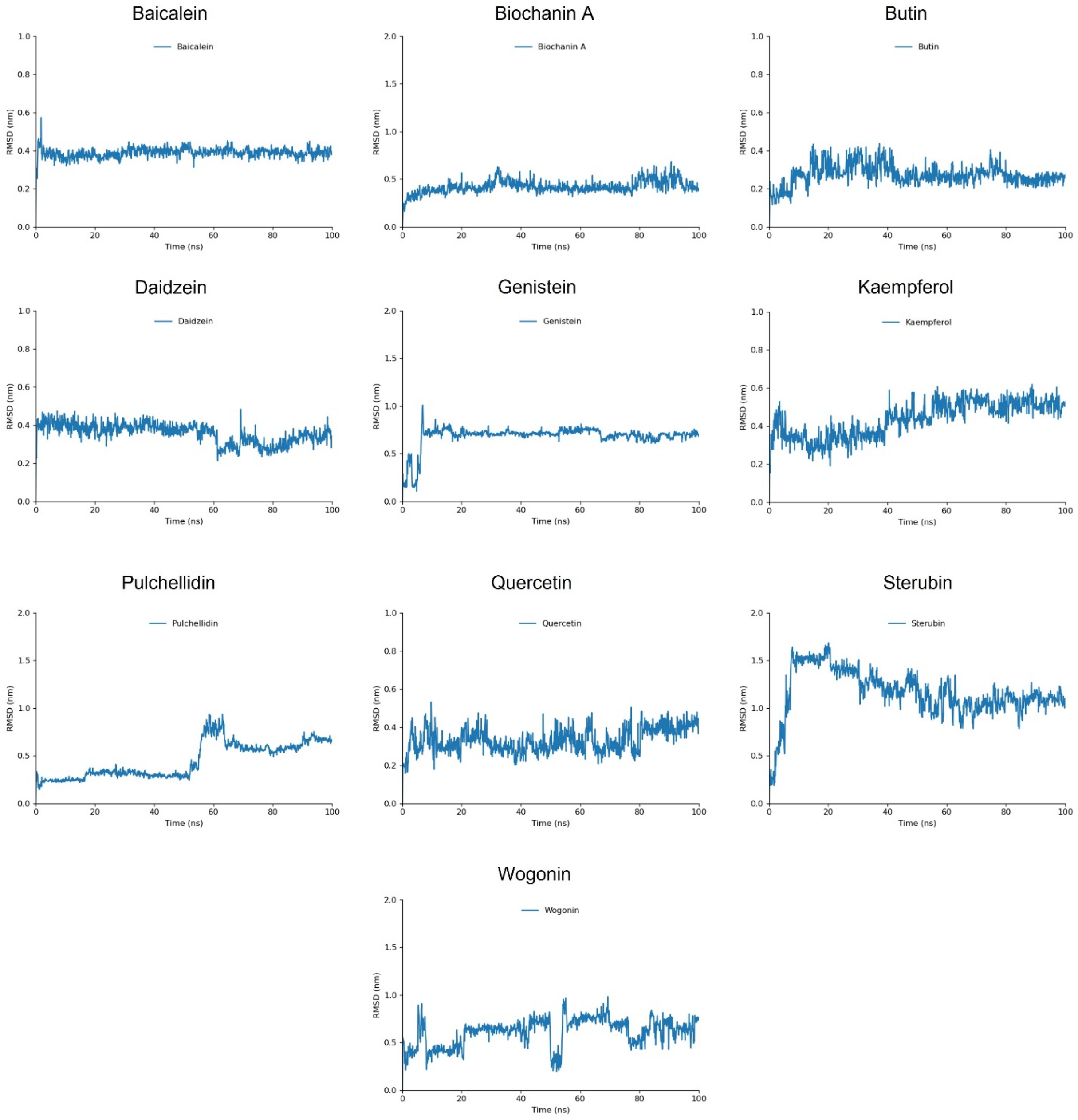

Figure S4. The individual RMSD plots of the simulated flavonoid compounds

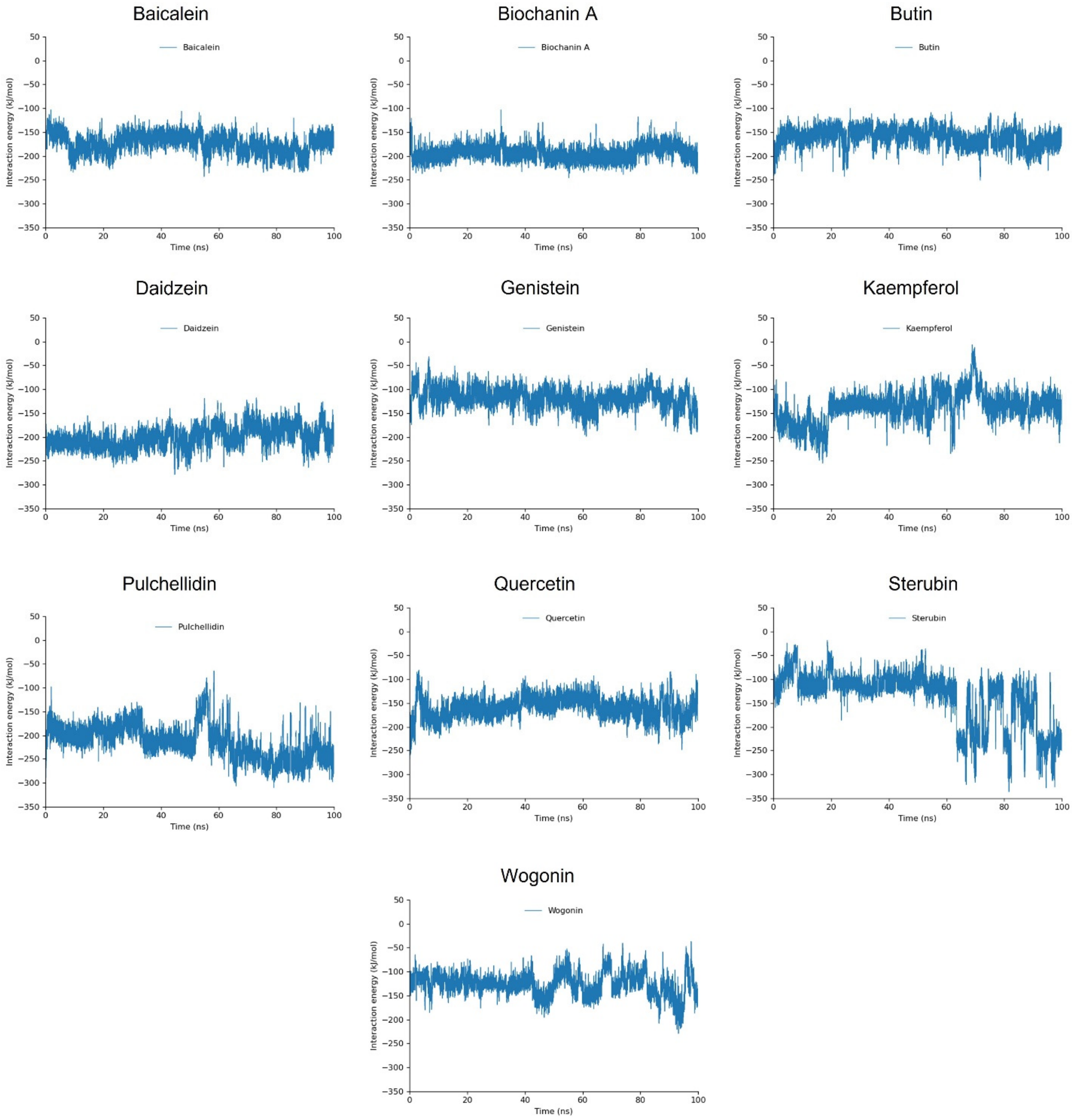

Figure S5. The individual interaction energy graphs of the simulated flavonoid compounds

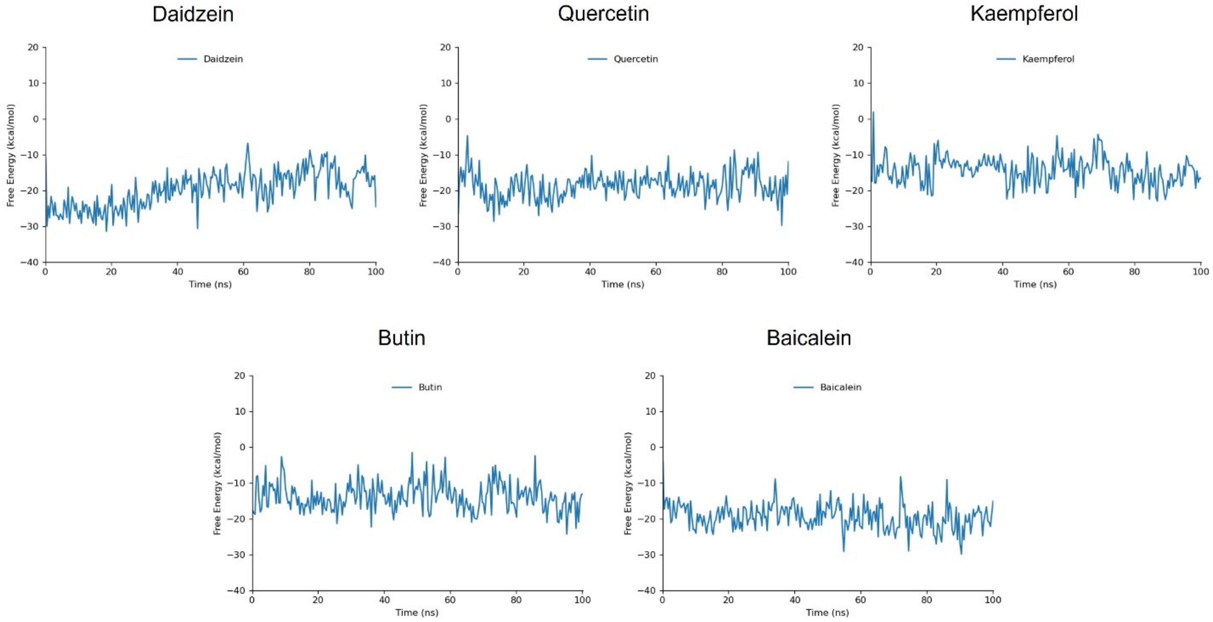

Figure S6. The individual calculated free energy graphs of top flavonoid compounds

1. Franko, A.; Berti, L.; Hennenlotter, J.; Rausch, S.; Scharpf, M. O.; de Angelis, M. H.; Stenzl, A.; Birkenfeld, A. L.; Peter, A.; Lutz, S. Z., Transcript levels of aldo-keto reductase family 1 subfamily C (AKR1C) are increased in prostate tissue of patients with type 2 diabetes. *J Pers Med* **2020,** 10, (3), 124.
2. Penning, T. M. J. C.-b. i., The aldo-keto reductases (AKRs): Overview. *Chem Biol Interact* **2015,** 234, 236-246.
3. Sirois, J.; Sayasith, K.; Brown, K. A.; Stock, A. E.; Bouchard, N.; Doré, M. J. H. r. u., Cyclooxygenase-2 and its role in ovulation: a 2004 account. *Hum Reprod Update* **2004,** 10, (5), 373-385.
4. Willard, L.; Ranjan, A.; Zhang, H.; Monzavi, H.; Boyko, R. F.; Sykes, B. D.; Wishart, D. S., VADAR: a web server for quantitative evaluation of protein structure quality. *Nucleic Acids Res* **2003,** 31, (13), 3316-9.
5. Yasir, M.; Park, J.; Han, E. T.; Park, W. S.; Han, J. H.; Kwon, Y. S.; Lee, H. J.; Chun, W., Computational Exploration of Licorice for Lead Compounds against Plasmodium vivax Duffy Binding Protein Utilizing Molecular Docking and Molecular Dynamic Simulation. *Molecules (Basel, Switzerland)* **2023,** 28, (8).
6. Yasir, M.; Park, J.; Chun, W., Discovery of Novel Aldose Reductase Inhibitors via the Integration of Ligand-Based and Structure-Based Virtual Screening with Experimental Validation. *ACS omega* **2024,** 9, (18), 20338-20349.
7. Rouzer, C. A.; Marnett, L. J. J. C. R., Structural and chemical biology of the interaction of cyclooxygenase with substrates and non-steroidal anti-inflammatory drugs. *Chem Rev* **2020,** 120, (15), 7592-7641.
8. Dwivedi, A. K.; Gurjar, V.; Kumar, S.; Singh, N. J. D. D. T., Molecular basis for nonspecificity of nonsteroidal anti-inflammatory drugs (NSAIDs). *Drug Discov Today* **2015,** 20, (7), 863-873.
9. Duggan, K. C.; Walters, M. J.; Musee, J.; Harp, J. M.; Kiefer, J. R.; Oates, J. A.; Marnett, L. J. J. J. o. b. c., Molecular basis for cyclooxygenase inhibition by the non-steroidal anti-inflammatory drug naproxen. *J Biol Chem* **2010,** 285, (45), 34950-34959.
10. Yasir, M.; Park, J.; Han, E.-T.; Park, W. S.; Han, J.-H.; Kwon, Y.-S.; Lee, H.-J.; Chun, W., Vismodegib Identified as a Novel COX-2 Inhibitor via Deep-Learning-Based Drug Repositioning and Molecular Docking Analysis. *ACS Omega* **2023,** 8, (37), 34160-34170.
11. El-Kabbani, O.; Darmanin, C.; Schneider, T. R.; Hazemann, I.; Ruiz, F.; Oka, M.; Joachimiak, A.; Schulze-Briese, C.; Tomizaki, T.; Mitschler, A.; Podjarny, A., Ultrahigh resolution drug design. II. Atomic resolution structures of human aldose reductase holoenzyme complexed with Fidarestat and Minalrestat: implications for the binding of cyclic imide inhibitors. *Proteins* **2004,** 55, (4), 805-13.
